# A longitudinal study of the role of fingers in the development of early number and arithmetic skills in children with Apert syndrome

**DOI:** 10.1111/joa.14111

**Published:** 2024-08-17

**Authors:** Caroline Hilton

**Affiliations:** ^1^ University College London London UK

**Keywords:** Apert syndrome, arithmetic, counting, early number, finger gnosis, fine motor skills working memory

## Abstract

This paper discusses a longitudinal study with children with Apert syndrome aged between 4 and 11 years. There has long been an interest in the role of fingers in the development of early number skills and arithmetic. As children with Apert syndrome are born with complex fusions of their fingers, they have to undergo several surgical procedures in order to obtain individuated fingers. This has implications for their finger mobility and finger awareness. It has been suggested that children with Apert syndrome have specific difficulties with early number and arithmetic activities. The findings from this study suggest that engaging children with Apert syndrome in activities that develop finger awareness (finger gnosis) and finger mobility (fine motor skills) may have a positive impact on their ability to engage with appropriate mathematics curricula at school. This is relevant to all those involved in the care of children with Apert syndrome and will be of particular relevance to those involved in early childhood and primary education. This study also provides new insights into the role of finger use in the development of skills and understanding in early number and arithmetic.

## Graphical abstract

There is evidence that finger gnosis and fine motor skills (FMS) can impact young children's progress in early number and mathematics activities. It is likely that the development of finger gnosis in children with Apert syndrome is atypical. Using interventions to develop finger gnosis and FMS, such as those illustrated (based on Gracia‐Bafalluy and Noel, 2008), children with Apert syndrome can be supported to better access the early mathematics curriculum alongside their peers.

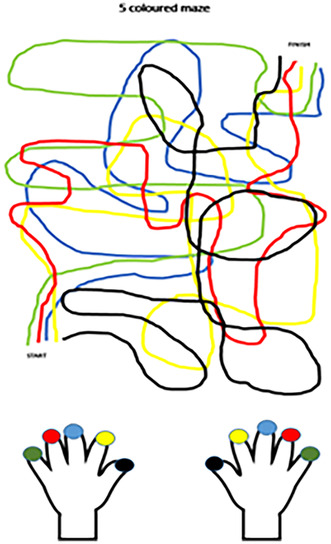

## 1 INTRODUCTION

There is much evidence to suggest that fingers play an important role in the development of early number concepts and arithmetic skills (for reviews, see Barrocas et al., 2020; Neveu et al., 2023). With this in mind, it is possible that children who have difficulties with finger gnosis and fine motor skills (FMS) are at greater risk for difficulties in developing skills in early number and arithmetic. The study discussed here focuses on the development of arithmetic skills in six children with Apert syndrome and explores the role of finger gnosis and FMS in their developing skills and understanding.

Apert syndrome has a birth prevalence of approximately 1 in 65,000 in Europe and North America and is equally present in boys and girls (Cohen Jr et al., 1992). Children with Apert syndrome are born with their fingers and toes fused (syndactyly) and with premature fusion of some of the sutures in their skull (craniosynostosis). Apert syndrome is caused by a mutation of the fibroblast growth factor receptor 2 (FGFR2) gene (Wilkie et al., 1995). The two mutations, S252W and P253R, account for 98% of those born with the syndrome (Stark et al., 2015).

Children with Apert syndrome often spend a lot of time in hospitals during their early years. As these children are born with complex syndactyly, they usually have several operations on their hands in order to achieve between four and five fingers on each hand (where “fingers” consist of all digits, including thumbs).

In terms of cognitive development, the literature suggests that there is significant variation in children with Apert syndrome (Lefebvre et al., 1986; Patton et al., 1988; Renier et al., 1996). It has been suggested that some of this variation may be due to factors such as speech and language development, visual impairments, hearing impairments and difficulties with FMS (Shipster et al., 2002), while others have suggested that the family environment might also have a significant impact (Yacubian‐Fernandes et al., 2004).

### 1.1 Hand anomalies in Apert syndrome

Hands in Apert syndrome involve unusually complex anomalies of both soft tissue and bony structures (Guero et al., 2004). The involvement of tendon, muscle, and neurovascular bundles presents a range of challenges for the paediatric hand surgeon, and children have to undergo several operations in order to achieve the best outcomes in terms of functionality and cosmetics (Guero et al., 2004).

Children with Apert syndrome usually have surgery on their hands over a number of years. Most protocols opt for surgery to be started at 6 months of age (Fearon, 2003; Zucker et al., 1991), while some centres prefer to start at 3 months (Guzanin et al., 2001) or 9 months (Fearon, 2003; Guero, 2005). Operations usually take place at 6 monthly intervals, with the aim of completing surgery by the time a child is 3 years old (Pettitt et al., 2017).

As children with Apert syndrome have very limited hand function prior to surgery—often unable to grip or grasp objects with one hand—surgical intervention is a priority in terms of functionality and access to typical activities (Zucker et al., 1991).

There are typically three types of hands in Apert syndrome, with varying degrees of fusion of the fingers. These have been categorised into three hand types (Upton, 1991):

- Type I—bony or cartilaginous fusion of the three middle fingers (index, middle and ring fingers).
- Type II—complex bony fusion of all the fingers (index, middle, ring and little fingers), with the thumb joined with skin but no bony fusion.
- Type III—complex bony fusion of all five digits.

Depending on the complexity of the fusion, children usually have a thumb and either three or four fingers following surgery. Due to the unusual joints, fingers in Apert syndrome do not bend, except at the knuckle and often in the final joint on the little finger. The thumb is usually short and radially deviated. Consequently, children do not tend to have a “normal” pinch grip, even after surgery (Taghinia et al., 2019). Although hand function after surgery seems to be similar for all hand types (Raposo‐Amaral et al., 2019), the resulting symphalangism (fusion of the interphalangeal joints) can make activities such as tying shoelaces and buttoning shirts very challenging.

### 1.2 Finger representation in Apert syndrome

Research on the somatosensory representation of fingers in Apert syndrome is very limited, and to date, it has only been possible to find one study on an adult who underwent finger separation (Mogilner et al., 1993). Prior to surgical separation of the fingers, the fingers were represented as one single digit (finger). Within a week after surgery, the area of the somatosensory representation had increased in size, and the fingers had more distinct cortical representations. However, even after 6 weeks, “the resulting hand area was smaller than normal and the organization was nonsomatotopic” (Mogilner et al., 1993, p. 3597). This suggests that the representation of the hand after surgery is unlike that of a typically developing hand. It is, however, known that the somatotopic maps can change in response to individual experiences and habits (Ogawa et al., 2019).

To try to understand what might be happening in the somotasensory cortex when fingers are separated in children with Apert syndrome, it is helpful to look at what is known about amputees. Weiss et al. (2000) explored what happened in a patient who had their middle and ring fingers amputated. They identified three distinct periods of change. The first change happened within the first 10 days and was very similar to that found by Mogilner et al. (1993). This finding was that there was a change in the demarcation of the finger representations. The second stage took several weeks or even months. This change saw significant changes in the connections being made within the somatosensory cortex. The final stage saw significant reorganisation based on how the fingers that were remaining were being used. This suggests that in Apert syndrome, individual finger use should be actively encouraged in order to establish more finely‐tuned changes in the somatosensory cortex.

This is very relevant if we now consider theories of embodied cognition which suggest that learning often takes place as a result of the sensorimotor interactions between an individual and their environment (Leung et al., 2011). This is of particular importance when we reflect on the role of finger gnosis and its possible impact on children with Apert syndrome.

### 1.3 Finger gnosis and Gerstmann syndrome

Finger gnosis is the ability to identify one's fingers without seeing them. In typically developing children, finger gnosis develops rapidly up to the age of 6 years and then continues to develop more slowly up to the age of 12 years (Strauss et al., 2006).

Gerstmann syndrome was first described in 1924. The syndrome is characterised by the presence of four conditions: finger agnosia (a lack of “finger sense”), acalculia, dysgraphia and left‐right disorientation. Finger agnosia was later described as an “elective disability for recognizing, naming, selecting, differentiating and indicating the individual fingers of either hand, the patient's own as well as those of other persons” (Gerstmann, 1940, p. 398).

It is thought that Gerstmann syndrome is the result of damage to the inferior parietal sulcus (Dehaene, 2011) or an issue with white matter fibre tracts in the parietal area (Rusconi et al., 2009).

Gerstmann (1940) observed that people with finger agnosia tend to make more finger identification errors with the three middle fingers than with the thumb and little finger.

### 1.4 Fingers and counting

Finger‐counting/montring activities, especially if practiced at an early age, can contribute to a fast and deep understanding of number concepts, which has an impact during the entire cycle of life by providing the sensory‐motor roots onto which the number concept grows. (Di Luca & Pesenti, 2011, p. 3)

“Finger‐montring” is the ability to show a particular amount with the correct number of fingers and without counting.

It has been shown that touching objects when counting supports pre‐school 4‐year‐old children to count the numbers of objects correctly (Alibali & DiRusso, 1999). This helps with understanding one‐to‐one correspondence and is less demanding on working memory because it helps with keeping track of the counted items. This suggests that fingers can provide an “egocentric sensory‐motor schema” (Rinaldi et al., 2016, p. 51) developed through regular repetition and practice.

In observational studies, fingers have been identified as playing an important role in supporting the development of understanding of our base‐10 number system (Anghileri, 2006; Fuson, 1982; Hughes, 1986). Fingers can represent both cardinality (the size of a set of objects) and ordinality (knowledge of the order of numbers—i.e. knowing that 7 comes before 8 in the sequence of natural numbers) (Domahs et al., 2008). It has been argued that fingers help to give meaning to these concepts (Sixtus et al., 2023). Fingers can be used to represent objects in calculations, thereby supporting the conceptual transition of numbers used to count real objects to numbers used as meaningful abstract operators (Anghileri, 2006; Hughes, 1986).

### 1.5 Using fingers in arithmetic calculations

Many studies over the last 20 years have found a relationship between both finger gnosis and FMS and the development of early number and arithmetic skills in typically developing young children (Newman, 2016; Noël, 2005; Penner‐Wilger & Anderson, 2013; Wasner et al., 2016). Recently, Krenger and Thevenot (2024), in a longitudinal study with children aged between 4 and 6 years, found that children who used their fingers to solve addition problems outperformed those that did not. The children were observed three times during 1 year, at 6‐monthly intervals. In addition, there is evidence that training in finger gnosis and FMS can positively impact arithmetic skills in typically developing 6‐ to 7‐year‐old children (Asakawa et al., 2019; Gracia‐Bafalluy & Noël, 2008). There is also evidence that interventions that develop finger use with children in Grade 1 (typically 6–7 years old) improve skills in solving addition and subtraction problems (Frey et al., 2024). In the study, this advantage was still present 9 months later for problems involving addition.

Soylu et al. (2018) have proposed reasons why the apparent relationship between finger gnosis and finger counting might exist. One suggestion is that better finger gnosis may be linked with better FMS and that both of these are needed for finger counting. Another suggestion is that finger gnosis is more directly associated with “number sense” which results in better finger counting. Perhaps sensorimotor activities support the ability to individuate fingers, which in turn support children to use their fingers to count. Michaux et al. (2013), in a study with adults, found that finger movements interfered with arithmetic processing, while foot movements had no effect. In addition, they found that the interference occurred more with addition and subtraction than with multiplication. This could be because children learn at a young age to use their fingers for addition and subtraction, and so these associations become more embodied. It could also be the case that multiplication facts tend to be in long‐term memory, and so are used more as retrieved facts.

Berteletti and Booth (2015), in a study with children aged between 8 and 13 years, found that the finger somatosensory and motor areas were activated during single‐digit subtraction tasks but not during multiplication tasks. They also found that the level of activation increased as the numbers in the calculations increased. In addition to this, there is the suggestion that “the negative correlations found between finger gnosis scores and activations in three visuospatial processing areas (i.e., left fusiform [and lingual gyri], and bilateral precuneus) during addition and subtraction….provide further neural evidence for the partially visuospatial nature of the link between finger gnosis and number processing” (Soylu et al., 2018, p. 118).

More recently, Artemenko et al. (2022) carried out a study with children in their first year of school (age range 6; 11–8; 4 years at the beginning of the study), with a control group and an intervention group. The intervention group received specific training in finger use during arithmetic activities for 18 sessions, lasting approximately 30 minutes each, during the first year of school. While this was a small study, the findings suggested the intervention group demonstrated increased activation in the sensorimotor cortex during single‐digit mental arithmetic, even when there was no specific finger movement observed. This suggests that even a short intervention can make a difference to the sensorimotor cortex.

What is the impact of finger use on the development of arithmetic knowledge and understanding? In a study of kindergarten children who had received no formal education, Jordan et al. (1992) found that when engaging with verbal calculations, finger counting was a strategy that separated the higher‐achieving middle‐income children from their lower‐income, lower‐achieving peers. Jordan, Kaplan, et al. (2008) later found that over time, the children from middle‐income families used their fingers less. During the same period of time, children from low‐income families continued to rely on finger use for arithmetic calculations. The delay of 2–3 years demonstrated by the lower‐income children suggests that it takes a long time for children to become confident enough to use known facts in arithmetic calculations and rely less on finger use. This evidence again supports the notion that finger use should be encouraged in early childhood education (Jordan, Kaplan, et al., 2008), but what does it mean for children with Apert syndrome, who only get to have individuated fingers during early childhood?

It is helpful to look briefly at how children develop their arithmetic skills. Fuson (1982) noticed that once young children begin to work on calculations using numbers less than 10, they have several strategies. She observed that when adding two one‐digit numbers together, children would usually count on mentally when the number being added was one or two. However, when this was greater than two, the children usually used their fingers to help them count on. Thompson (1995) had similar findings with older children, between 6 and 8 years of age. This is further evidence that “finger gnosia may serve as a mechanism to offload working memory demands, helping children to accurately represent quantities above the subitizing range, which in turn support arithmetic processing” (Costa et al., 2011, p. 9).

The question now might be whether there are other strategies that could help in the same way. Fuson and Secada (1986) explored this with children aged between 8 and 10 years. They used two interventions—one with fingers and one with dot patterns. One group of children was shown how to use their fingers (by tapping their fingers on the table) to help with solving arithmetic problems, while the other group was shown how to use dot patterns (i.e. drawing the number of dots in a problem). The group that used fingers continued beyond the intervention stage, but the group that used dot patterns did not use them spontaneously after the intervention stage. Fuson and Secada (1986, p. 256) suggested that “most children spontaneously related counting on with finger patterns to their schemas of addition and thus counted on with finger patterns to solve addition word problems”.

In a recent scoping review of the literature on the relationship between finger use and the development of arithmetic skills in children and adolescents, Neveu et al. (2023) identified one of the challenges as the fact that much of the research comes from two distinct research domains: mathematics education and cognitive psychology and neuroscience. This has resulted in contradictory findings and much debate within this field of study. Even so, Neveu et al. found that 66.7% of the studies exploring the relationship between finger gnosis and arithmetic skills supported the notion that the development of finger gnosis can support the development of arithmetic skills in children. However, Neveu et al. (p. 25) suggest that:

Although these studies have investigated the direct influence of finger‐based strategies or finger sensorimotor skills on arithmetic skills, they have not addressed how finger‐based strategies are related to finger sensorimotor skills or how this relation influences children's arithmetic skills.

It has, however, been suggested that, in typically developing children, not only can finger gnosis predict attainment in arithmetic, but that finger representations become better defined as children get older (Reeve & Humberstone, 2011).

A number of studies have also explored the relationship between working memory and finger use in arithmetic tasks. Geary et al. (2004), for example, in a study of children with mathematical difficulties (MD) (defined as those with mathematical reasoning scores below the 30th percentile), found that 6–7‐year‐old children with MD were more likely to use finger counting strategies to solve addition problems than their typically developing peers. While this was associated with lower scores on a test of working memory, it should be noted that of the 21 children in the study, 14 also scored below the 30th percentile in reading. This suggests that these findings may be the result of a complex interplay of different factors.

More recently, Dupont‐Boime and Thevenot (2018) compared the strategies used by 6‐year‐old children when solving simple addition problems. They found that finger use and proficiency in addition calculations were highly correlated. They also found that children with high working memory capacities used more efficient strategies (counting on the largest addend) than the children with lower working memory capacities (who used a counting all strategy).

### 1.6 Working memory and arithmetic

As has been suggested, working memory is often associated with proficiency in arithmetic tasks (see Zhang et al., 2022, for a detailed review). Working memory is best described as a person's ability to hold and manipulate information mentally over short periods of time (Gathercole & Alloway, 2008). Poor working memory is often associated with low attainment in mathematics (Geary, 2011). Pickering and Gathercole (2001) use a model of working memory with three components. These components are: the phonological loop (PL); the visuo‐spatial sketchpad (VSSP); and the central executive (CE).

The PL uses auditory information and is associated with spoken and written language. Information is held in the PL for approximately 2 seconds, unless it is consciously rehearsed. The VSSP stores information that is non‐verbal and is either visual (e.g. colour or shape) or spatial (e.g. movement or position).

The CE is responsible for the more complex aspects of working memory. The CE manages and organises activities such as the flow of information, planning and retrieval of information from long‐term memory.

In studies involving 6‐, 7‐ and 14‐year‐old children in the United Kingdom, a correlation was found between children's working memory and their attainment in mathematics (Gathercole & Alloway, 2008). Gathercole and Alloway (p. 54), suggest that there are several reasons for the children's specific difficulty with arithmetic:

First, working memory overload in the individual activities designed to develop numeracy skills will result in frequent errors and task failures, impairing the incremental process of acquiring basic number skills and knowledge, and slowing down the child's rate of learning. Second, mental arithmetic is heavily dependent on working memory….it requires not only the storage of arbitrary numerical information, but also the retrieval and application of number rules that may not yet have been securely learned.

Hitch et al. (2001) proposed that speed of processing might also play a role. They argued that if a person processes information more quickly, they are less likely to forget. This suggests that a child who processes information quickly will not need to “hold” the information for as long as a child who processes information more slowly.

There are also differences in the challenges children with particular disabilities and learning difficulties face. For example, it has been suggested that children with motor coordination difficulties tend to have stronger PL than VSSP skills, while children with language impairments tend to have stronger VSSP than PL skills (Gathercole & Alloway, 2008). Cowan et al. (2005), on the other hand, found that in their study of 7‐ to 9‐year‐old children with specific language impairments, they had poor VSSP in addition to their poor PL skills. It may be the case that the populations of children had other differences that impacted their working memory skills that have not been documented in the literature.

What is important to note is that adults and children rely on working memory, even for simple arithmetic tasks (Cragg et al., 2017). Moreover, in a meta‐analysis of 46 studies, a correlation was found between working memory and arithmetic skills and attainment (Zhang et al., 2022). Overall, verbal working memory was more strongly correlated with arithmetic than visuospatial working memory, especially for addition and subtraction. There were no differences with tasks that involved either written or mental tasks of arithmetic. However, while the role of verbal working memory seems to decline with age, the role of visuospatial working memory persists.

### 1.7 The development of arithmetic skills in children with Apert syndrome

Children with Apert syndrome usually have a complex profile. This may include, but is not restricted to: limited finger mobility; a visible difference (or disfigurement); hearing impairments and visual impairments. The literature on Apert syndrome has tended to focus predominantly on surgical management of the syndrome, with far less emphasis on cognitive, psychological and social aspects of development (Hilton, 2017).

There is little focus on the experiences of children with Apert syndrome in school, specifically on aspects related to approaches to teaching and learning. With regard to mathematical development specifically, only one study was found (Sarimski, 1997) that explored attainment in this curriculum area. In this study of nine children with Apert syndrome, seven of the children for whom there were results scored lower in tests of arithmetic than they did in tests investigating perceptual and verbal skills. In a later study carried out by Fearon and Podner (2013), parents of children with Apert syndrome reported that their children experienced greater difficulty with mathematics than tasks involving verbal and reading skills.

## 2 THE CURRENT STUDY

The purpose of the study was to begin to address the gap in the literature on how children with Apert syndrome develop skills in early number and arithmetic. The research questions were:

- What strategies do children with Apert syndrome use to help them solve numerical problems involving arithmetic operations?
- Do the children's hand anomalies impact the range of strategies available to them?

### 2.1 Methods

The longitudinal study took place over 2½ years. The researcher worked with 10 children, all of whom lived in the United Kingdom. The children were visited in their homes prior to starting in‐school observations and assessments. Due to the variation in the children's ages and their learning differences, a qualitative approach was used, based on an interpretivist paradigm. This paradigm is particularly appropriate for small‐scale studies, focusing on gaining an understanding of meanings and actions and investigating behaviours that are taken for granted (Cohen et al., 2007). For example, in this study, there was a focus on individual children, whose educational profiles were based on a set of norms and expectations that are value‐laden, although these may not be made explicit. In order to gain a deeper understanding of the children's development in this study, an approach that attempted to explore factors that might be impacting their attainment and access to the curriculum was required. In addition, as children with Apert syndrome have very complex profiles (due to the impact of features such as visual and hearing impairments, low expectations and teasing), an approach that allowed for these differences seemed appropriate. This more individualised approach has been recommended for clinical studies with children with complex profiles. The suggestion is that clinical guidance should be developed by linking observational studies with more evidence‐based approaches (Hayward et al., 2016).

A case study approach was adopted in order to explore each case in depth over the 2½ years of the study. Case studies can provide insights into individual strengths, weaknesses and differences in ways that are not possible with quantitative approaches where ther is a greater reliance on group averages. Although it is not always possible to make generalisations from case studies based on the methods applied in quantitative approaches, they do provide evidence from which theoretical propositions can be deduced (Yin, 2009). It has been suggested that frequently, “in experimental studies, results are presented on group level, with neither individual differences nor the children's reasoning made explicit. Thus, external validity at group level may be high while ecological validity at the level of preschool or individual children is often low” (Palmér & Björklund, 2024, pp. 2–3).

During the school visits, the children were interviewed one to one and observed in class. For the one to one interviews with the children, a “clinical interview” approach was used (Ginsburg, 1981) in order to gain insight into what the children were thinking and doing. This approach is based on the work of Piaget, who used clinical interviews when exploring children's understanding of concepts. A clinical interview goes beyond what is possible through standardised tests and attempts to explore children's cognitive processes and understanding as well assess competence.

Overall, in order to provide data from a range of perspectives, a range of tools were used. Following the data collection process, analysis was carried out using thematic analysis (Braun & Clarke, 2022).

### 2.2 Procedures

All the children were initially visited in their homes. This provided the opportunity to interview the parents and meet the children. The school visits usually took place for a whole day. This made it easier for the researcher to fit in with the school routines and also maximised time spent with the children and staff. The one to one clinical interviews lasted between 20 min and an hour, depending on factors such as the age of the child, the amount of time available, how well the child was coping and their preferences. For consistency and reliability, the questions used for the one to one interviews were based on existing assessments that had been used in previous studies. The children's finger gnosis was assessed on some of the visits. The interviews were audio recorded and transcribed as soon as possible after the school visits. Staff were also interviewed during the school visits.

### 2.3 Participants

The children in the study all attended mainstream or special schools in the United Kingdom. All participants were recruited via the UK charity *Headlines Craniofacial Support* (a charity that supports individuals and families affected by craniosynostosis). Ten families responded with children between 4 and 9 years of age. Details of the children, in terms of their age at the start of the study, number of fingers and type of school attended, are provided in Table 1.

**TABLE 1:** Age of children at the beginning of the study, number of fingers and type of school attended.

Child (age at start)	Number of finger (right hand/left hand)	Type of school attended
C1 (4 years)	5/5	Mainstream school
C2 (5 years)	5/5	Mainstream school
C3 (5 years)	5/5	Special school
C4 (5 years)	5/4	Mainstream school
C5 (5 years)	4/5	Mainstream school
C6 (7 years)	5/5	Mainstream school
C7 (8 years)	5/5	Mainstream school
C8 (9 years)	5/5	Mainstream school
C9 (9 years)	4/4	Mainstream school
C10 (9 years)	5/4	Mainstream school

### 2.4 Ethical issues

The study was approved by the Ethics Research Committee of the Institute of Education, University of London (now University College London). Written consent was provided by the parents/guardians of the children, and the children also assented to being part of the research project. Parents/guardians and children were advised that they could opt out of the study at any time. Permission was obtained from the head teachers of the schools and from the staff involved. All the analysed data were pseudonymised and the data collected were stored securely.

### 2.5 Materials

During the one to one interviews, the following areas of mathematical development and understanding were explored:

- Early counting skills and an informal understanding of arithmetic (Gelman & Gallistel, 1978; Hughes, 1986).
- “Number sense”, using activities adapted from Jordan, Glutting, et al. (2008) number sense screening tool. These included questions involving counting and comparing, more formal arithmetic questions and word problems involving addition and subtraction.
- Number knowledge was explored using activities adapted from the “Number Knowledge Test” (Griffin & Case, 1997). This test is designed for children from 3 to 10 years of age.
- The children's ability to subitise (i.e. the ability to instantly recognise how many items there are without counting) and compare quantities.
- Numerical Operations and Mathematical Reasoning components of the “WIAT‐ II” (Wechsler, 2005), as these are commonly used for assessing children in the United Kingdom.

The children's working memory was assessed, as poor working memory is often associated with poor arithmetic skills (Zhang et al., 2022). This was assessed using the Working Memory Test Battery for Children (WMTB‐C) (Pickering & Gathercole, 2001).

Finger gnosis was assessed using an approach based on Gracia‐Bafalluy and Noël (2008). For this assessment, each hand was tested separately. The hand that was being tested was covered with an A4 piece of paper so that the child could see their hand. The researcher then touched individual fingers and recorded the results. When a finger was touched, the paper was removed so that the child could identify the finger that had been touched. This was different from the method used by Gracia‐Bafalluy and Noël. In their study, the hand remained covered and the child had to identify the finger that had been touched on a graphic image of a hand. The one‐finger touches were repeated on the other hand. The second part of the assessment was to touch two fingers at the same time. Again, this was done one hand at a time, and the results were recorded.

## 3 ANALYSIS

As the study took place over 2½ years, some analysis took place during the study period, allowing for an iterative process. Consequently, each child's development could be explored in some depth, and the impact of any interventions could be explored. A process of reflexive thematic analysis, based on Braun and Clarke (2022), was adopted. Reflexive thematic analysis allows for the fact that the researcher brings a level of subjectivity to the analytic process, but views this as a resource as they engage with the data and the theory in order to develop their interpretation.

After an initial familiarisation with the data, they were systematically coded and the themes highlighted. Through a process of further refinement, the final themes were identified.

### 3.1 Results

As a case study approach was adopted, it is helpful to view three cases in some detail in order to explore the processes and methods used by the children at different observation points during the study. This will be followed by a general discussion in relation to the whole group of children.

Each child in the study had between eight and 10 fingers altogether, so for some of the children, there was a challenge when trying to “make 10”. The children were creative in the ways they used their fingers to do this. Some of the strategies that the children used are explored below. In the descriptions of the strategies, the fingers are numbered from one to four or five on each hand, where number one is the thumb and the rest of the fingers are numbered in canonical order.

The development of C3, C5 and C7 at key points will be discussed here. These children have been chosen because of the different ways in which they engaged with finger gnosis training. C3 did not engage in any formal finger gnosis training; C5 engaged in formal finger gnosis training at school following the method proposed by Gracia‐Bafalluy and Noël (2008); and C7 reportedly used her fingers as a very young child and, importantly, played the piano at home. All the children had hearing impairments (all conductive hearing loss) and visual impairments (these differed). They all wore hearing aids (some in‐ear and some bone conduction) and glasses. Although the aids would have helped, they did not give the children “normal” hearing and vision. None of the children could bend their fingers at the interphalangeal joints, and it was hard for them to bend their fingers at the metacarpophalangeal joint. As mentioned previously, the clinical interviews (Ginsburg, 1981) with the children lasted between 20 and 60 min, depending on their engagement and interest. The interviews were always ended if the children wanted to go back to class or seemed to be struggling to focus.

In the extracts that follow, the interviewer is identified by “I” and children are all referred to as “she”, to assure confidentiality.

#### 3.1.1 C3 (5 years old at the beginning of the study, five fingers on each hand)

The first observation of C3 was in class. The children in the class were asked to “show” finger combinations (finger montring) for numbers from one to five. C3 could not put individual fingers down to “show” the numbers. She could show “one” with her index finger, but to make “two” and “three”, she had to hold down the “unwanted” fingers with the other hand. With activities involving clapping and stamping feet for a given number, C3 had no difficulty engaging, but tasks involving finger montring seemed to involve much more effort. At the end of the lesson, the children were asked how many fingers they had altogether. C3 counted all her fingers, counting each finger with the index finger of the other hand, starting with the thumb on her left hand and counting on canonically. When she had counted all her fingers, C3 announced, “I've got ten fingers!”

During the first visit, C3 engaged very well in the clinical interview with the researcher. In the activities involving plastic counters/tokens, C3 touched the plastic counters/tokens as she counted them with the index finger on her right hand. When five plastic counters/tokens were presented, C3 counted them one at a time (“One, two, three, four, five”) and also held up five fingers on her right hand when she had finished. This suggests that she understood the cardinal value of the set.

##### First observation +4 months

The teacher had begun to try to include activities to develop finger montring in mathematics lessons. The mathematics lesson began again with a “show me” activity with the numbers one to five. For “Show me five digits,” C3 showed her full left hand. For “Show me four,” C3 put her thumb in. For “Show me three”, C3 seemed to struggle, as she was not able to move her index finger or little finger down like the teacher. C3 had similar difficulties with number two. For “Show me one”, however, C3 was able to individuate her index finger and lower the other fingers.

During the clinical interview C3's finger montring was explored in a bit more depth:

I Can you show me five fingers?

[C3 puts up one hand her left hand]

I Can you show me 10 fingers?

[C3 puts up both hands]

I Can you show me two fingers?

[C3 struggles to hold up her index and middle fingers on her left hand]

I Can you show me…

C3 One…

C3 One finger?

[C3 isolates her index finger on her left hand by lowering the other fingers]

I Can you show me three fingers?

C3 Three

[C3 tries to isolate 3 fingers on her left hand, by holding the index middle and ring fingers]

I Can you show me four fingers? Four…Show me four…Five [as C3 puts up her whole left hand]…Where's four?

[C3 puts down her thumb to show 4 fingers]

C3 There it is

An attempt was made to assess C3's finger gnosis using a method based on Gracia‐Bafalluy and Noël (2008). C3's right hand was covered, and her index finger was touched. When asked to show which finger had been touched, C3 showed her whole hand. The activity was modelled by the researcher, but this did not seem to help. The assessment was repeated four more times, touching different fingers on her right hand, but each time C3 was asked which finger had been touched, she showed her whole hand. It is hard to know whether C3 did not understand what was being asked or whether it felt to her as if her whole hand had been touched. This is reminiscent of the findings of Mogilner et al. (1993), discussed earlier.

##### First observation +12 months

C3 was first observed in class. The lesson began with another finger montring activity. C3 was reluctant to participate and appeared distracted. The next series of activities involved clapping and foot stamping. This time, C3 was very engaged.

##### T + 16 months

On this visit, the researcher attempted the WMTB‐C (Pickering & Gathercole, 2001). C3 struggled with understanding what was required for some of the tasks (see Table 2).

**TABLE 2:** Combined WMTB‐C and WIAT‐II standard scores.

Child	Digit recall	Block recall	Backward digit recall	WIAT‐II mathematical reasoning	WIAT‐II numerical operations
	Phonological loop	Visuospatial sketchpad	Central executive		
C1	80	98	84	87	63
C2	122	—	—	83	78
C3	127	98	—	78	66
C4	144	86	134	103	113
C5	142	108	125	99	103
C6	77	110	—	82	82
C7	145	118	107	93	85
C8	81	86	80	53	58
C9	111	109	85	57	60
C10	138	118	107	82	88

This was the first time that C3 had been able to engage with the finger gnosis assessment. She got all the single touches correct but struggled when two fingers were touched. When two fingers were touched, she either identified fingers that had not been touched or *two fingers that were next to each other*.

##### First observation +26 months

During the clinical interview, C3 was given the opportunity to use her fingers or plastic counters/tokens to solve arithmetic problems. On each occasion, she chose to use plastic counters/tokens.

C3 is interesting because she did not engage in any finger gnosis training, either formal or informal. She did not tend to use her fingers to support arithmetic activities, and at the last assessment, she had quite undeveloped finger gnosis. In earlier attempts to assess her finger gnosis, C3 struggled. It was not clear whether this was because she did not understand the question or because she had no idea which finger was being touched. While some attempts had been made by one of her teachers to develop finger montring, this was not something that C3 was generally seen using, except when prompted. As C3's finger gnosis was developing, it seems possible that the mismatch between the visual representations of fingers used for finger montring conflicted with the sensory experience when the fingers were touched. This could create a situation of confusion for a child. Throughout the whole study, C3's teachers noted that she was working on single‐digit addition and subtraction. She could solve arithmetic problems that were couched within real‐life scenarios or with objects that could be seen, but struggled with the more abstract symbolic problems (e.g. 3 + 2).

#### 3.1.2 C5 (5 years old at the beginning of the study, 5 fingers on the left hand, 4 fingers on the right hand)

C5 regularly played games at home (e.g. Snakes and Ladders, dominoes) and actively engaged in counting during daily activities, such as counting out cutlery at meal times. C5's parents were concerned about C5's development in number and addition, and her class teacher described her understanding of number as “poor”.

During the first interview, C5 was able to add and subtract up to two:

I Right if I put the six counters back in here [as I put the counters back into the bag]. How many's in here?

C5 Six

I OK if I take one out [as I take a counter out of the bag] how many are in here now?

C5 Five [very confident]

I Should we check?

C5 Yeah

I [I tip the counters out] Were you right?

C5 One, two, three, four, five [touching the counters as she counts] yeah

I If I put the five back in here [as I put the counters into the bag]

C5 Yeah

I How many are in here? [as I shakes the bag] [pause] How many did I put in?

C5 Five

I If I take out two [as I takes out 2 counters] how many will be left?

C5 Three [very confident]

I Well done. You did those really quickly. Do you do a lot of things like this at home?

C5 Yeah

I With counters?

C5 No with buttons

C5 was able to add on three to a starting number less than five, without using her fingers or any other concrete manipulatives.

C5's finger gnosis was assessed during this visit. On the left hand, with one finger identification, C5 was able to correctly identify fingers 1, 2 and 5, but consistently mixed up fingers 3 and 4. When two fingers were touched, C5 was able to identify the correct fingers for the combination of 1 and 5 on her left hand, but seemed to pick any fingers when other combinations were touched. On the right hand, C5 could correctly identify fingers when one finger was touched. With two fingers, C5 could correctly identify her thumb when it was touched, but was not consistent with any other fingers.

##### T + 4 months

At this time, C5's teacher was still very worried about her understanding of number. The teacher suggested that C5 “…knows a lot of number facts, but finds it harder to apply her knowledge.” That was an interesting comment that was not supported by the way that C5 approached the problems during the clinical interview.

I If you had four chocolates

C5 Yeah

I You can write things down if you want to [as I passes C5 some paper]….if you had four chocolates and I gave you three more…

**FIGURE 1:**
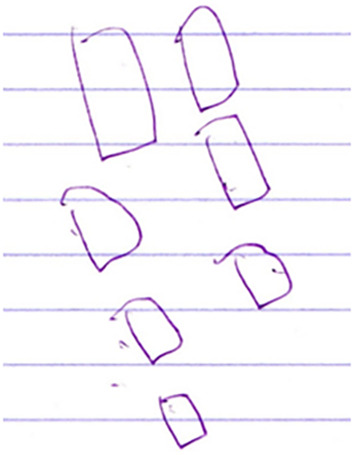
C5's drawing of “four chocolates and three more”.

[C5 draws four rectangles and then three more, see Figure 1].

I How many would you have altogether?

C5 One, two, three, four, five, six, seven [pointing to the images as she counted]

I Do you want to write that down?

**FIGURE 2:**
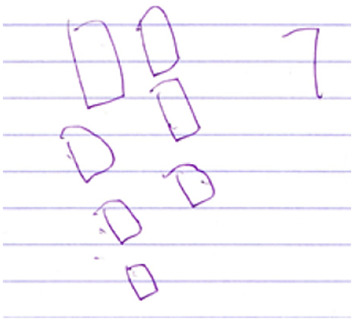
C5 adds the answer “7”.

[C5 writes 7 next to the rectangles, see Figure 2].

This was later followed by a task involving counting back.

I OK…What number comes four numbers before sixty? Four numbers before sixty

C5 Fifty‐five…no, fifty‐nine, fifty‐eight, fifty‐seven, fifty‐six

C5 did not use her fingers to help with this and yet she was able to keep track.

##### First observation +7 months

On this occasion, the WMTB‐C (Pickering & Gathercole, 2001) was carried out. As can be seen in Table 2, C5 had many strengths. This may go some way to explain why she was able to complete numerical tasks mentally without the use of fingers or concrete manipulatives.

C5's teacher had been working with C5 on the finger gnosis training as outlined by Gracia‐Bafalluy and Noël (2008) since we last met. Rather than using coloured stickers, C5's teacher suggested C5 should number each finger. Consequently, when the finger gnosis assessment was carried out, C5 was able to say which fingers she thought had been touched by naming the finger.

When one finger was touched, C5 was correct with every trial on her right hand, but mixed up fingers 3 and 4 on her left hand. When two fingers were touched, C5 made errors with every pair and even made some errors when finger 1 was touched. When fingers 1 and 5 on her left hand or 1 and 4 on her right hand were touched, C5 could identify one of the fingers correctly. C5's finger gnosis seemed very similar to the previous assessment, which had taken place seven months earlier.

##### First observation +10 months

This time with the finger gnosis assessment, C5, it was very successful with both hands and with one and two finger touches. On her left hand, she made a few mistakes when fingers 3 and 4 were touched, but on other trials, she identified them correctly. This was a significant change from the last visit.

##### First observation +17 months

What was interesting on this occasion was the choice of method that C5 used. Some of the questions from previous interviews were used, but the methods C5 used to solve these questions were different. Some examples have been provided below.

I If you had four chocolates

C5 Yeah

I And someone gave you three more

C5 Yeah

I How many would you have altogether?

C5 Four, five, six, seven [counting on her fingers, touching the fingers on her left hand, in canonical order, with the index finger on her right hand]

However, when adding two, she could do it without her fingers and could explain her working

I OK ummm how much is two plus four?

C5 Eight…six

I Which one, eight or six

C5 Six

I Why is it six?

C5 Because six is before…six is after four

I Yes. How many numbers after four is six?

C5 Two [said with no hesitation]

For subtraction, C5 used her fingers to help her count back

I Lovely. How much is eight take away six?

C5 Seven, six, five, four, three, two [using her fingers].

I Right, can you do thirty‐six? You might want to write this down…thirty‐six take away eighteen [C5 writes down 36–18 as it is read out to her]

C5 Thirty six take away ten is twenty six

I Yep

C5 And twenty‐six, twenty‐five, [counts correctly down to eighteen using her fingers. On her right hand, C5 counted both sides of her middle finger, thereby making this hand (with only four fingers) have five items to count]

[C5 writes = 18 on the end of the calculation].

When C5's finger gnosis was assessed, she was correct on every trial on both hands with one finger and two finger touches.

What was worthy of note on this visit was C5's confidence and ability to articulate what she was doing. She demonstrated flexible use of number relationships, number facts and more informal methods. In terms of visible strategies, the most distinct one was her increased use of fingers in problem‐solving, which possibly provided her with the “mental space” to focus on the methods she was using (Costa et al., 2011).

#### 3.1.3 C7 (8 years old at start of the study, five finger both hands)

C7's parents reported that she enjoyed playing the piano. They said that she definitely used her fingers for number work when she was younger, but that she used them much less now she was older. They were concerned about her progress in mathematics and had appointed a tutor to help at home. They were happy to encourage her to use her fingers.

On the first visit to C7's school (2 months after the home visit), she demonstrated confidence with numbers up to 20. For example:

I And what comes two numbers after seven?

C7 [short pause] Nine.

I What number comes two numbers before eighteen?

C7 Sixteen?

I Uh huh. And two numbers after eighteen?

C7 Twenty?

I Yeah. How are you working those out?

C7 Ummm I'm just counting in twos

I You're counting in twos. OK, fantastic. Do you use your fingers at all to help? [The researcher had noticed that C7 seemed to be touching her leg with her fingers]

C7 Yeah

And later:

I Kisha has six pennies. Peter takes away four of her pennies. How many pennies does Kisha have left?

[C7 takes time]

I Kisha has six pennies. Peter takes away four of her pennies. How many pennies does Kisha have left?

[C7 takes time]

C7 Two

I How did you work that one out?

C7 I just used my fingers and counted back [This time touching the table]

I OK…from?

C7 Did you say six?

I I did say six…that's very good

C7 Yeah, I counted back from six

##### First observation +5 months

On this visit, the WMTB‐C (Pickering & Gathercole, 2001) was carried out. C7 scored very highly. C7's finger gnosis was also assessed using the model based on Gracia‐Bafalluy and Noël (2008). On both hands, C7 was quickly able to identify fingers 1, 2 and 5, whether they were touched on their own or in conjunction with another finger. However, on both hands, when fingers 3 and 4 were touched, C7 was not able to identify the correct finger (i.e. finger 3 for finger 4 or finger 4 for finger 3).

##### First observation +11 months

On this visit, C7 continued to use her fingers to help with questions. What is noticeable this time, is C7's confidence and speed of calculation. For example:

I Right. Five ducks were swimming in a pond

C7 OK

I Three flew away and then two more came to swim [C7 touched her fingers on the table, as she worked this out]

C7 Four [answered before the question had been completed]

I How many were left? How did you work that out?

C7 Five take away three is two add two is four [spoken very confidently]. Easy

On this occasion, when C7's finger gnosis was assessed, she was almost perfect on every trial with one finger and then two fingers, on both hands. C7 made one mistake when fingers 1 and 3 were touched on her right hand, but was able to answer correctly when the trial was repeated.

##### First observation +16 months

On this visit, C7's finger gnosis was assessed on her left hand only, as C7 has recently had an operation on one of the web spaces on her right hand. C7 was very quick and accurate with all one‐finger touches and two‐finger touches.

This time, C7 was a bit more confident with modelling problems than she had been before. For example (Wechsler, 2005, Mathematical Reasoning, Item 30):

Robert has 6 stones.

Together Robert and Max have 15 stones.

How many stones does Max have?

C7 very quickly provided the answer “Nine”. When asked how she had worked this out C7 said, “I realised that six and ten was sixteen, so six and nine would be fifteen”.

This response seems to show progress from the pure counting‐based methods used previously. In this example, C7 appears to be using a derived strategy (Thompson, 2000) based on her knowledge of number combinations and place value (“I realised that six and ten was sixteen”) and her understanding of n‐1 principles (“so six and nine would be fifteen”) (Dowker, 2012). This requires an integrated understanding of both the principles of counting and the procedure of counting (Baroody & Ginsburg, 1986), together with an integration of the process of counting and the concept of number (or numerosity) (Gray & Tall, 1991). It seems that C7 no longer relied completely on counting and was able to use known facts to derive new ones.

From observations, C7 was becoming more confident with her understanding of arithmetic and problem solving. During the last visit, C7 demonstrated less use of fingers, together with a useful set of known number facts from which she was able to derive others. In other words, she was beginning to see patterns and relationships in mathematics that had not been obvious to her before.

With C5 and C7, as their finger gnosis and finger use increased, so too did the range of strategies they could use. They could both use their fingers to support their calculations, but they could also do calculations without their fingers. It seemed that their models or representations of the arithmetic relationships had become more sophisticated, and the strategies that they had at their disposal were more varied and adaptable.

### 3.2 Summary of findings for all cases

Having discussed three cases in some depth, the following overarching themes emerged. These will be summarised in relation to the findings for the whole group of children.

- Working memory
- Subitising and comparison
- Knowledge of counting
- Strategies for solving arithmetic problems
- Different ways of using fingers
- Changes in finger gnosis

When the parents and school staff were interviewed at the beginning of the study, they all voiced their concerns about the children's progress in mathematics, which they felt was more of a problem than in other areas of the curriculum. Many of the teachers also said that they did not encourage the children to use their fingers to help with solving problems in mathematics because they were worried that the children would find this physically challenging. All the parents and school staff were informed about the literature on the role of finger gnosis. Many of the schools used the activities from the literature, while others used activities of their own. In all the cases where finger use was actively encouraged and maintained, there was a change in the ways in which the children solved simple arithmetic problems.

#### 3.2.1 Working memory

It is useful to look at the working memory scores for the children, as this seems to correlate with the children's skills in arithmetic (Zhang et al., 2022). Table 2 shows the children's scores in the working memory assessments and their scores in the mathematical elements of the WIAT‐II. As can be seen, the children have quite uneven profiles. In nearly all cases, the children's standardised working memory scores are above their standardised scores in the mathematics assessments. This suggests that working memory was not a factor that was hindering their skills and understanding of early number and arithmetic.

#### 3.2.2 Subitising, comparison and counting

All the children were able to perceptually subitise up to at least three. To do this, plastic counters/tokens were used, as these were manipulatives with which the children were already familiar. There was less consistency with conceptual subitising (e.g. the ability to recognise dice patterns), as this was more dependent on the children's experience of playing games at home. All the children were able to correctly compare piles of plastic counters/tokens and say which was the larger or smaller. They were also able to say which number was bigger when the question was asked either verbally or in written form. All the children had knowledge of the number sequence and were able to correctly identify the sets of objects that were presented during the clinical interviews. These were all important skills to assess, especially given the relationship between counting on and counting back and skills in addition and subtraction (Steffe et al., 1983).

#### 3.2.3 Strategies for solving arithmetic problems

We have already seen how the strategies used for solving arithmetic problems by C3, C5 and C7 changed over time and how finger use gave two of them a way to offload aspects of tasks so that they could access a range of informal strategies to support arithmetic problem solving (Costa et al., 2011: Crollen & Noël, 2015). A further example of the impact of finger use when the working memory demands of a task are high can be seen with C4. Below is an extract from an interview with C4 at 5 years of age.

I If I put these three into this envelope [puts in three counters]

C4 Yeah

I And I put one more in [puts one more counter into the envelope]. How many will there be in the envelope now?

C4 [pause] four

I How did you know that?

C4 I counted in my head

Now look at the extract below with the same child.

I How much is three and two?

C4 Five

I Well done…how much is four and three?

[12 second pause]

C4 Four and three?

I Yes four and three

C4 Seven

I And how much is two and four?

[23 second pause]

I Should we leave that one?

C4 Yeah

While C4 was working with numbers that she was able to work with mentally, she could answer all the questions. However, it seems that as soon as the questions took her beyond her working memory capacity, she had no strategy to help with the calculation.

These are reminiscent of Fuson (1982), who found that when children were unable to work out answers to problems mentally, they usually used their fingers in order to help them keep track of the count. This was not a strategy that C4 was familiar with, and so what might have been an intuitive strategy for a typically developing child was not yet available to C4.

By the time C4 was 7 years old, there had been a significant change in the way that she engaged in arithmetic problem solving. C4's parents and some of the support staff that worked with C4, had been encouraging the use of fingers to support mathematical problem solving sporadically during the study. More recently, C4's teaching assistant had been paying particular attention to her use of fingers, as she was concerned about C4's attainment in mathematics. The extract below is between C4 and her teaching assistant (TA), after about 3 months of finger awareness activities.

TA How would you work out twelve plus fifty‐four?…How do we work it out?

C4 I don't know

TA Which number would you work out first….which number do we normally pick when we're adding?

C4 Twelve?

TA [Name] which number do we normally start with the bigger number or the smaller number?

C4 Big

TA So which number are you going to start with?

C4 Fifty‐four

TA And then what will be your next step if you have fifty‐four

C4 Fifty‐five, fifty‐six, fifty‐seven [continues to count correctly to sixty‐six, using all the fingers on her right hand and using her left hand to keep track of the tens]

C4 counted on both hands for numbers up to 10 (adding on the missing “10” when she had counted all nine fingers), touching each finger with the index finger of the other hand. For the addition of numbers greater than 10, C4 counted the ones on her right hand (which had five fingers), while keeping track of the 10s on her left hand (which had 4 fingers) so that she could continue counting. As she counted, C4 would touch her fingers with the index finger of the hand that was not being counted.

The strategy modelled here shows understanding of the base‐10 number system and the use of fingers for keeping track of the tens and for counting on.

This extract again highlights the key role that finger use can play in supporting children to be successful in arithmetic problem solving. The difficulty highlighted when C4 was 5 years old did not seem to be with understanding the mathematical concepts but rather with the strategy used. The use of fingers as a tool enabled her to offload elements of the calculations as she worked through the problem.

#### 3.2.4 Changes in finger gnosis and finger use

Gracia‐Bafalluy and Noël (2008) found that after 8 weeks of finger gnosis training with kindergarten children, there was an impact that lasted for at least 1 year. For the children in this study, the finger gnosis training had to be continued for at least 6 months to obtain lasting results. What was interesting, as has been demonstrated by C3 and C4, was that once children became confident with finger use, they used their fingers in quite complex ways and not just to count on in ones. This demonstrates an understanding of mathematical concepts and the use of fingers as a sophisticated tool to aid calculation.

The children who had fewer than 10 fingers created their own strategies for making “10”. This was not only a useful strategy but also highlighted the children's understanding of the importance of being able to “see” 10 when using their fingers.

## 4 DISCUSSION AND CONCLUSIONS

This paper describes the first longitudinal study exploring arithmetic development and the role of finger gnosis in children with Apert syndrome. The approach used provided an opportunity to explore the children's individual differences and the changing strategies they used when engaging with arithmetic problem solving. The findings suggest that it is unusual for children with Apert syndrome to spontaneously use their fingers to help with arithmetic calculations. This is not unusual in young children (3–5 years of age), especially for counting (Roesch et al., 2024). Significantly, Roesch et al. (p.13) found that

prompted finger counting as well as prompted finger number gesturing turned out to be significant predictors of verbal counting, cardinal number understanding, and basic arithmetic in 3‐ to 5‐year‐olds over and above influences of age and general cognitive ability.

Lê et al. (2024), in a study exploring the use of fingers versus the use of manipulatives in high‐achieving Vietnamese 3–5 –year‐olds, found that there was no advantage to the use of fingers over the use of manipulatives. However, given that finger gnosis is rapidly developing at this point, makes it less surprising that young children may rely less on finger use. It may take time for finger use to become more embedded as a resource that provides sensory motor and visuospatial input (Soylu et al., 2018). Lê et al. found that there was a relationship between children who had high working memory capacities and their number skills. What the current study seems to show is that when working memory is at capacity, children can get stuck if they do not use some sort of representation, such as fingers or some other manipulative.

While fingers may not be the only way to access numerical understanding, the embodied nature of learning that takes place when fingers are used and the fact that we always have our fingers with us make it reasonable to propose that some sort of finger awareness training should be provided for all children with Apert syndrome, from an early age. This may benefit not only their number skills but also other aspects of the development of sensory and fine motor skills.

Rusconi et al. (2014) suggested that the developmental trajectory for finger gnosis is longer than for awareness of other parts of the body. This is due to the fact that finger awareness needs to be very fine‐tuned in order for fingers to be as dextrous as possible. For children with Apert syndrome, this will have consequences for the amount of time that is required for full finger awareness to develop. Further research needs to be done to find out what this means for the development of the somatosensory cortex and how this compares with a typically developing population.

As has already been discussed, children who underperform in arithmetic tasks, often have associated weaknesses in their working memory (Zhang et al., 2022). In this group of children, working memory did not seem to be a factor in the children's underachievement in arithmetic skills. Moreover, Dupont‐Boime and Thevenot (2018) found that 6‐year‐old children with high working memory capacities were more likely to use their fingers to accurately solve tasks involving addition. This was not generally the case with the children described in the present study, suggesting that the factors that might impact finger use in typically developing young children, such as seeing it modelled at home (for example, C4's twin sister typically used her fingers to help with arithmetic calculations, while C4 did not) or high socio‐economic status (Jordan, Kaplan, et al., 2008), are not sufficient for children with Apert syndrome. This is likely due to the atypical development of the sensorimotor system in children with Apert syndrome during the process of finger separation.

**FIGURE 3:**
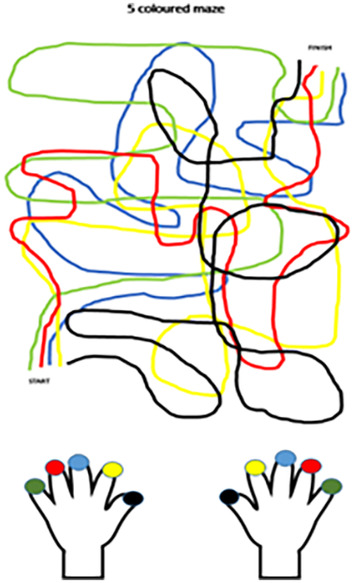
Example of maze task with five colours. The coloured stickers on the fingers indicate which path is to be traced with each finger.

A possible suggestion to explain why children with Apert syndrome may be reluctant to use their fingers in the early years of primary school may relate to Soylu et al. (2018) suggestion that the visuospatial processing areas are involved during addition and subtraction. The children discussed in this study, who had poor finger gnosis, demonstrated an initial reluctance to use their fingers to support arithmetic calculations. If the developmental trajectory for finger gnosis requires time and practice as suggested by Rusconi et al. (2014) and if the representation of fingers in the brains of children with Apert syndrome is nonsomatotopic (Mogilner et al., 1993), the visual image of fingers when finger montring may conflict with the sensorimotor experience for some time after the fingers have been separated. For example, if a child isolates two fingers, say fingers 2 and 3, to support with a calculation, but when they touch the fingers, it feels like fingers 2 and 4, this confusion could lead to a sense of uncertainty and thereby a disincentive to continue with this strategy. For this reason, activities such as those suggested by Gracia‐Bafalluy and Noël (2008) are very appropriate because they are solely finger gnosis activities and are not related to finger use for the purpose of arithmetic problem solving (see Figure 3 for an example of this). Over time, it is possible that through engaging with these activities, the visual map and the sensory map come together, thereby making it more likely that children will use their fingers to support them in solving numerical problems.

What is the role of FMS? Pitchford et al. (2016) argued that FMS are associated with attainment in mathematics in young children. This idea was developed further by Fischer et al. (2022). Previous studies had suggested a link between young children's number skills and their FMS, especially dexterity (Barrocas et al., 2020). What is often lacking in these studies is a clear definition of FMS. Fischer et al. (2022) tried to address this by separating manual dexterity from finger agility. Manual dexterity was defined as the ability to manipulate objects in tasks such as bead threading, while finger agility was defined as the ability to intentionally move one's fingers, as in finger tapping activities. Dexterity was not found to be correlated with finger montring or finger counting. However, finger agility was found to be closely associated with finger counting, and finger gnosis was found to be closely associated with finger montring. For finger montring the authors suggest that, although there is a need for motor control, the need for finger awareness as well as visual control has a greater impact. For finger counting, there is a greater need to have finger agility in order to extend one finger after the other when, for example, counting out the correct number of fingers for a task. This theory has implications for all children, but especially children with Apert syndrome. Additionally, the tasks in finger gnosis training activities, such as those suggested by Gracia‐Bafalluy and Noël (2008) help to develop finger individuation (or agility), as well as developing finger gnosis through sensory input, in order to, for example, trace a path on a maze (see Figure 3).

This study provides insights that are useful for those working with children with Apert syndrome and their families. The findings suggest that children with Apert syndrome would benefit from support to develop their finger gnosis and FMS in a systematic way, as soon as possible after the surgical release of their fingers. It also informs the literature on the role of finger gnosis and FMS in the development of early number and arithmetic skills and understanding by providing some detail in terms of what progression looks like and how using fingers serves to encourage creativity and flexibility in the application of arithmetic knowledge and understanding.

## 5 LIMITATIONS

As there is a very small population of children with Apert syndrome in the United Kingdom, this study had to be small‐scale. Although there are two variants of Apert syndrome, this study did not explore the differences between the two types. There were many variations in the children's experiences in school and at home that could not be controlled for. This led to some differences in the ways that the interventions were carried out in school, as well as differences in terms of support and advice that the children received from professionals. The children went to different hospitals within the United Kingdom. Most of them had their specialist medical care in the specialist centres in England, but some went to hospitals in the jurisdictions in which they lived. Consequently, the children experienced different treatment protocols, which may have impacted decisions concerning hand surgery. It would be useful if future studies could explore the benefits of particular interventions to develop finger gnosis and finger training in children with Apert syndrome.

## AUTHOR CONTRIBUTIONS

The paper is solely the work of the author.

## CONFLICT OF INTEREST STATEMENT

None.

## Data Availability

New data have been shared.
